# Effect of soil bioremediation on soil microbial community structure aimed at controlling tobacco bacterial wilt

**DOI:** 10.1007/s00253-023-12753-4

**Published:** 2023-09-27

**Authors:** Yanxia Liu, Han Li, Xiang Li, Heng Zhang, Jingwei Zhu, Yu Peng, Guangjun Sun, Jian Xu

**Affiliations:** 1 Guizhou Academy of Tobacco Science (Guizhou Provincial Academician Workstation of Microbiology and Health), Guiyang, 550000 China; 2 Guizhou Tobacco Corporation of CNTC, Guiyang, 550000 China

**Keywords:** Tobacco bacterial wilt, Integrated control measures, Microbial community structure, Functional genes, Microbial networks

## Abstract

**Abstract:**

Rebuilding soil healthy microbiota is very important for preventing bacterial wilt. A 3-year-long field trial was conducted in China as follows: T1 (conventional fertilization), T2 (T1 + liming), T3 (T1 + bioorganic fertilizer), and T4 (T2 + bioorganic fertilizer). Fluorescence quantitative PCR and high-throughput sequencing were employed to study the dynamics of *Ralstonia solanacearum* population, microbial community, and network organizations between bacteria and quality-related variables. After 3 years of bioremediation, the control efficacy of tobacco bacterial wilt reached 61.30% and the occurrence delayed by approximately 40 days in T4, which had the highest tobacco yield and output value. The pathogen population of T4 remained below 10^6^ copies/g soil during the entire growth period. Role-shifts prevailed among the network members. Microbes were unipathically associated with variables in T1 but multiplex in T4. In conclusion, soil bioremediation rebuilds a healthy soil microbiota and forms a more interactive and relevant micro-system, thus effectively controlling tobacco bacterial wilt.

**Key points:**

*• This is the first time to effectively bio-control tobacco bacterial wilt in practical production in China, as well as to high-efficiently use the organic waste, thus promoting the organic cycling of the environment.*

*• Soil bioremediation can effectively control soil-borne disease by rebuilding soil healthy microbiota and reducing abundance of pathogenic bacteria, thereby to prevent the soil borne disease occurrence.*

*• After the soil remediated, microbes associated with soil and tobacco characteristics changed from unipathical to multiplex, and the keystone species play different roles compared with the original soil, thus signifying the complexity of multi-species interactions and achieving a closely relevant micro-system, which was ecologically meaningful to the environment.*

**Supplementary Information:**

The online version contains supplementary material available at 10.1007/s00253-023-12753-4.

## Introduction

Tobacco bacterial wilt, caused by *Ralstonia solanacearum* E.F. Smith f. sp. *nicotianae*, is one of the most destructive bacterial soil-borne diseases and affects tobacco production in Guizhou Province, China (Liu et al. 2015). Many traditional strategies such as tillage management are not always effective, since *R. solanacearum* can survive in soils for a long time (King et al. 2008). Crop rotation and disease-resistance breeding are proved effectively in controlling soil-borne disease. However, crop rotation is often unrealistic in China due to the limited amount of land available for tobacco growing, and the breeding of disease-resistant varieties often leads to low yield and quality of tobacco leaves (Liu 2014). In addition, the application of traditional chemicals (bactericides) to control bacterial wilt has been shown in some cases to be minimally effective and at the same time has resulted in a negative impact on the environment health risks to tobacco consumers (Liu et al. 2013). More recently, the Chinese government proposed a “double reduction policy”; therefore, biocontrol and bioremediation have been considered a promising management strategy (Zhang et al. 2011; Wang et al. 2013a). At present, research on the use of antagonistic bacteria to control bacterial wilt is mainly confined to the laboratory, and poor control effects have been examined in pot or field conditions in practical production (Jiang et al. 2017).

Antagonistic bacteria rarely colonize the root after soil application because native microbes aggressively outcompete invasive microbes and the applied strains are poorly adapted to the soil environment. Thus, it is crucial for biocontrol to increase the colonization ability of antagonistic bacteria. Bioorganic fertilizer, in which antagonistic bacteria are secondarily fermented with organic fertilizer, can control bacterial wilt by improving soil microbial ecology, suppressing pathogens and increasing plant resistance (Abawi and Widmer 2000; Li et al. 2009). Organic fertilizers are one of the most effective soil amendments because they not only improve soil quality, but also provide the antagonistic microbes with nutrients, thus ensuring their survival and allowing them to reach a population number sufficient to disease control. Zhao et al. (2014b) found that *Paenibacillus polymyxa* SQR21 enhanced bioorganic fertilizer (BIO), decreased the population of *Fusarium oxysporum* in soil rhizosphere, and effectively controlled watermelon *Fusarium* wilt to a certain extent. Nevertheless, the agricultural environments are variable and complex. Single measure like microbial agent application and soil pH regulation can only change a single element in a short time among many pathogenic factors. Due to the unique conditions of agricultural environments and the limitation of single prevention technology, there is no single measure for bioremediating severely diseased field soil until now (Chen 2014). Wei et al. (2017) suggested that a single biocontrol agent performed unstable under field conditions. The integrated measures for biocontrol can eliminate the cask effect caused by a single prevention technology and become an effective approach to the prevention of soil-borne disease (Wang et al. 2013b; Liu et al. 2014).

In this field trial, integrated control measures including soil pretreating with lime or application of bioorganic fertilizer were applied in a severely tobacco bacterial wilt–infected field for 3 years in Guizhou Province, Southwest China. We explored the effect and soil microbiota in the rhizosphere soil. The most significant effect was observed after 3 years of bioremediation. The combined use of lime and bio-organic fertilizer can obtain the best disease control efficacy with the delayed occurrence simultaneously, as well as the highest tobacco yield and output value. The results of this research can lead to a better understanding of the biocontrol of tobacco bacterial wilt and provide a foundation for effective biocontrol mechanisms.

## Materials and methods

### Development of disease-control bioorganic fertilizer

The antagonistic strain LX5 (*Bacillus licheniformis*) was isolated from previously healthy tobacco rhizosphere soil and stored in the China General Microbiological Culture Collection Center as CGMCC No. 8266 (Li et al. 2017a, b). The purified LX5 strain was incubated in Luria–Bertani medium at 28 °C, 170 rpm for approximately 36 h and subsequently centrifuged at 8000 rpm × g at 4 °C for 10 min. The precipitate was re-suspended in the same volume of sterilized distilled water. And then the strain suspension was secondary fermented with organic compost, consisting of pig manure, rapeseed cake fertilizer, and vinasse organic fertilizer (1:1:1 *w/w/w*). Nearly 5% (*v/w*) of the strain LX5 culture was inoculated into organic fertilizer and fermented at 40–45% moisture for approximately 7 days, during which time the fertilizer was turned over three times per day (Wu et al. 2014).

Counts of strain LX5 were determined by real-time PCR (Almeida et al. 2018) and repeated three times. After solid fermentation, the population of strain LX5 reached 1.91 × 10^9^ copies/g of fertilizer (Table S1). The bioorganic fertilizer contained 33.8% organic matter, 4.30% amino acids, 4.20% N, 2.26% P_2_O_5_, and 1.08% K_2_O.

### Field trial design and soil properties

Tobacco seeds of Yunyan 85 (*Nicotiana tabacum* L.), a variety susceptible to bacterial wilt, were used in the field experiment. Tobacco in this field has been cultivated for more than 10 consecutive years, and the bacterial wilt disease incidence was 100%.

A 3-year-long field trial was conducted in Changshun (26.03°N, 106.45°E), Guizhou Province. This farmland decided to no longer cultivate tobacco plants due to the severe disease incidence. There were four treatments used in the field trial. T1: 65 g of tobacco-specific chemical fertilizer (N:P_2_O_5_:K_2_O = 10:15:25) was applied per plant; T2: soil was pretreated with 600 kg/hm^2^ of lime to adjust the pH value 20 days before transplanting, and then 65 g per plant of tobacco-specific chemical fertilizer (N:P_2_O_5_:K_2_O = 10:15:25) was applied. T3: 54.5 g of tobacco-specific chemical fertilizer and 15 g of bioorganic fertilizer were applied per plant; T4: soil was pretreated with 600 kg/hm^2^ of lime to adjust the pH value 20 days before transplanting, and then 54.5 g of tobacco-specific chemical fertilizer and 15 g of bioorganic fertilizer were applied per plant. The same nutrient elements were adjusted in all treatments with the application of elemental fertilizers. Each treatment had four blocks randomly arranged with 120 tobacco plants per replicate. The field covered an area of 0.05 hm^2^.

The soil was classified as yellow soil, and the parent materials were Triassic limestone and weathered sand shale. The initial soil properties were pH 5.45 (1:1 soil water ratio), 1.91 g·kg^−1^ of total nitrogen, 0.10 g·kg^−1^ of total phosphorus, 1.69% of total potassium, and 30.28 g·kg^−1^ of organic matter. The determination of soil texture referred to soil agrochemical analysis (Bao 2000). Soil organic matter, available nitrogen (mg·kg^−1^), available phosphorus (mg·kg^−1^), available potassium (mg·kg^−1^), pH, etc. were measured and analyzed after tobacco harvest and respectively used heating potassium dichromate volumetric method, alkaline hydrolysis diffusion method, HCL-H_2_SO_4_ method, NH_4_OAc leaching flame photometry, and soil:water = 1:2.5.

### Disease incidence and analysis of quality characteristics

Observations of wilt incidence and severity were made every 10 days from 30 to 90 days after transplanting. Tobacco leaves were collected when harvested to determine the tobacco yield and the output value, which were calculated after leaves had been flue-cured (Zhang et al. 2012).

The disease incidence index (DII) and the wilt control efficacy were calculated according to the following equations (Liu 2012):

$${\text{Disease incidence index }}\left( {{\text{DII}}} \right) = \left[ {\sum\limits_{i = 1}^{n} {(Si \times Pi)/(n \times Sn)} } \right] \times 100$$

where *n* is the total number of tobacco plants in each treatment, the disease severity *S* is expressed by a grading method by dividing the onset into several levels from mild to severe, *Sn* is the highest rating on the disease severity scale, *P*_*i*_ is the percentage of wilt incidence, and *S*_*i*_ is the severity scale of the wilting symptoms used to evaluate disease development on individual plants.

$${\text{Control efficacy = }}\left[ {{{\left( {{\text{CK}}_{{{\text{DII}}}} - {\text{T}}_{{{\text{DII}}}} } \right)} \mathord{\left/ {\vphantom {{\left( {{\text{CK}}_{{{\text{DII}}}} - {\text{T}}_{{{\text{DII}}}} } \right)} {{\text{CK}}_{{{\text{DII}}}} }}} \right. \kern-0pt} {{\text{CK}}_{{{\text{DII}}}} }}} \right] \times 100\%$$

where CK _DII_ is the disease incidence index of the control treatment (*P*_*R*_) and *T*_DII_ is the disease incidence index of the individual treatment.

The pretreatment of tobacco leaves adopted the normal temperature extraction method of n-hexane (Li et al. 2016). The qualitative and quantitative analysis of nicotine, megastigmatrienone, solanone, and other substances in leaf samples was measured by high-performance liquid chromatography-gas chromatography-mass spectrometry. HPLC conditions: the mobile phase was dichloromethane, the flow rate was 0.25 mL/min; the injection volume of tobacco extract was 10 μL; the column temperature was 30 °C. LC-GC/MS interface conditions: The LC-GC interface was On-column and retention gap technique, and the pre-column was a deactivated elastic quartz capillary, with size of 5 m × 0.53 mm. The solvent evaporation temperature was 40 °C, and the flow rate of helium gas (99.999% purity, China) was 80 mL/min.

After the LC transfer was over, the evaporation was continued for 0.7 min. GC/MS conditions: the instrument was Agilent 6890/5975 gas chromatography-mass spectrometer (Agilent, USA). The separation column was DB-5MS, with size of 30 m × 0.25 mm × 0.25 μm. The carrier gas was high-purity helium. The column flow rate was 1.2 mL/min (constant flow mode). The temperature program of the GC oven was as follows: 40 °C for 14 min, then increased to 290 °C at 4 °C/min, and held for 5 min. The GC/MS transfer line temperature was 280 °C, the MS ion source was 230 °C, the quadrupole was 170 °C, and the mass scanning range was 45–350 amu. Mass spectrometry identification was performed by parallel search of NIST08 and WILEY 6.0 spectral libraries. The internal standard method was used for quantification, assuming a relative correction factor of 1.

### Sample collection and DNA extraction

Rhizosphere soil was collected 90 days after transplanting. Tobacco plants were gently uprooted from the trays and shaken gently to remove all but the most tightly adhered soil. The soil was placed in 10 mL of sterile water and sonicated for 15 min to facilitate soil release (Carlsen et al. 2012). The DNA was extracted in triplicate using a Soil DNA Isolation Kit (Omega Biotek, USA) and then diluted with distilled sterile water to 1 ng μL^−1^. The quality and concentration of extracted DNA were determined with a NanoVue spectrophotometer (GE Life Sciences, USA).

### Soil pathogen and antagonistic bacteria detection

Fluorescence real-time PCR assays to quantify DNA parameters of *R. solanacearum* and *Bacillus licheniformis* were conducted using the primer pairs flicF-flicR and F1-R1, respectively, and amplified in a StepOne Plus Real-time PCR System (ABI, USA). Real-time PCR amplification reactions were carried out using the SYBR® Premix Ex Taq™ (TaKaRa Bio Technology Co., Ltd., Japan). The amplification procedure followed that described by Liu et al. (2014) and Li et al. (2013).

According to the real-time PCR results, the amplification efficiencies of *R. solanacearum* and *B. licheniformis* were 91% and 100%, respectively. The standard curve of *R. solanacearum* was *C*_t_*R* =  − 3.564*C*_0_*R* + 38.744, while the standard curve of *B. licheniformis* was *C*_t_*B* =  − 3.322*C*_0_*B* + 37.209. The counts of *R. solanacearum* and *B. licheniformis* were calculated according to the *C*_t_ value on the basis of the standard curve (Liu 2012).

### Library construction and metagenomics sequencing

Soil DNA was fragmented to an average size of about 300 bp using Covaris M220 (Gene Company Limited, China) for paired-end library construction. Paired-end library was prepared by using TruSeq™ DNA Sample Prep Kit (Illumina, San Diego, CA, USA). Adapters containing the full complement of sequencing primer hybridization sites were ligated to the Blunt-end fragments. Paired-end sequencing was performed on Illumina HiSeq4000 platform (Illumina Inc., San Diego, CA, USA) at Majorbio Bio-Pharm Technology Co., Ltd. (Shanghai, China) using HiSeq 3000/4000 PE Cluster Kit and HiSeq 3000/4000 SBS Kits according to the manufacturer’s instructions. All the raw metagenomics datasets have been deposited into NCBI Sequence Read Achieve database (PRJNA897573).

### Genome assembly, prediction, taxonomy, and functional annotation

De bruijn-graph-based assembler SOAPdenovo (http://soap.genomics.org.cn, Version 1.06) was employed to assemble short reads. K-mers, varying from 1/3 to approximately 2/3 of reads length, were tested for each sample. Scaffolds with a length over 500 bp were retained for statistical tests; we evaluated the quality and quantity of scaffolds generated by each assembly and finally chose the best K-mer which yielded the minimum scaffold number and the maximum value of N50 and N90. Then, scaffolds with a length over 500 bp were extracted and broken into contigs without gaps. Contigs were used for further gene prediction and annotation.

Open reading frames (ORFs) from each metagenomic sample were predicted using MetaGene (http://metagene.cb.k.u-tokyo.ac.jp/). The predicted ORFs with length being or over 100 bp were retrieved and translated to amino acid sequences using the NCBI translation table.

The α-diversity metrics (i.e., Chao1, Simpson’s, and Shannon index) were calculated based on OTU numbers. BLASTP (Version 2.2.28 + , http://blast.ncbi.nlm.nih.gov/Blast.cgi) was employed for taxonomic annotations by aligning non-redundant gene catalogs against NCBI NR database with *e*-value cutoff of 1e^−5^. Each colored brand represents the average value of each taxonomic abundance according to the color scale. Cluster of orthologous groups of proteins (COG) for the ORFs annotation was performed using BLASTP against eggNOG database (v4.5) with an *e*-value cutoff of 1e^−5^. The KEGG pathway annotation was conducted using BLASTP search (Version 2.2.28 +) against the Kyoto Encyclopedia of Genes and Genomes database (http://www.genome.jp/keeg/) with an *e*-value cutoff of 1e^−5^.

### Network analysis

Network analysis was performed using the Molecular Ecological Network Analyses Pipeline (http://ieg2.ou.edu/MENA/main.cgi). More information on theories, algorithms, pipeline structure, and procedures can be found in the references (Zhou et al. 2011; Deng et al. 2012).

### Data analysis

The data obtained were statistically analyzed using Microsoft Excel 2010, SPSS Base Ver. 13.0 (SPSS Inc., Chicago, USA), and Design-Expert 8.0. The data were subjected to a one-way ANOVA, and the means were tested with the Duncan multiple range test at *P* ≤ 0.05.

## Results

### Physicochemical properties of tobacco leaf and soil

After harvest, the soil physi-chemical properties were improved neither by lime nor single bioorganic fertilizer (Table S2), but by the integrated employment of both. The effect on pH adjustment from lime was obviously efficient. However, due to its violent reaction, it will damage the ecological environment of the soil to a certain extent. The contents of nitrogen, phosphorus, and potassium, especially ammonium nitrogen, were decreased with different gradients. In T2 treatment, the ammonia nitrogen content reduced by 17.6%, 29.4%, and 31.2% compared with T1, T3, and T4, respectively, since alkaline environment was very unfriendly to ammonia nitrogen. The nutrient contents from T4 treatment were significantly higher than T1 treatment, except for total nitrogen, indicating that the integrated application of lime and bioorganic fertilizer ameliorated soil physi-chemical properties efficiently.

The contents of leaf nicotine and total nitrogen in T1 were higher than those in T4 treatment (Table S3), while the total sugar, reducing sugar, potassium, megastigmatrienone, solanone, and norsolandione were significantly lower in T1 than those in T4 treatment. Single adjusted rise in pH brought insignificant changes in leaf quality. Compared with control, the nicotine and total nitrogen reduced 5.71% and 8.37%, and total sugar, potassium, megastigmatrienone, solanone, and norsolandione increased 7.02%, 15.00%, 3.87%, 11.47%, and 8.48%, respectively.

### Tobacco bacterial wilt control efficacy using the integrated measures

In 2017, even though the integrated control measures were applied to the soil, tobacco bacterial wilt spread extensively across the entire field 53 days after transplanting. Thus, differences in control efficacy among treatments were not significant (Table 1). At the end of the 2018 tobacco growing period, the wilt incidence was 100% in T1, whereas in T4, it was significantly lower than that of control (Fig. S1). Across the entire 2018 growing period, the control efficacy of T4 was significantly higher (*P* ≤ 0.05) than that of T2 and T3; 90 days after transplanting, the control efficacy of T4 had increased by 78.1% and 18.2% compared with T2 and T3, respectively. In 2019, the occurrence of tobacco bacterial wilt in T3 and T4 was delayed by approximately 40 days compared with the control. Sixty days after transplanting, the control efficacy in T4 was significantly higher than that of T2 and T3; 90 days after transplanting, it was 6.26- and 1.99-fold as high as in T2 and T3, respectively.

**Table 1:** The effect of integrated control measures on the control efficacy of tobacco bacterial wilt

Year	Treatment	Control efficiency in field experiments (%)						
		30 days	40 days	50 days	60 days	70 days	80 days	90 days
2017	T1	-	-	-	-	-	-	-
	T2	3.1 ± 0.8c	0 c	0 b	0a	0a	0a	0a
	T3	5.1 ± 1.9b	2.1 ± 0.5b	0 b	0a	0a	0a	0a
	T4	9.2 ± 3.9a	4.2 ± 1.2a	0.9 ± 0.2a	0a	0a	0a	0a
2018	T1	-	-	-	-	-	-	-
	T2	56.3 ± 5.4b	54.2 ± 2.7b	53.1 ± 6.2c	50.3 ± 5.1c	46.2 ± 2.2c	40.3 ± 0.8c	40.2 ± 1.7c
	T3	70.6 ± 7.1a	72.1 ± 4.9a	70.3 ± 2.0b	68.5 ± 3.7b	63.2 ± 1.7b	60.7 ± 1.7b	60.6 ± 5.3b
	T4	76.5 ± 4.2a	77.3 ± 6.2a	75.0 ± 1.2a	73.8 ± 1.8a	73.9 ± 3.8a	71.6 ± 4.3a	71.6 ± 1.5a
2019	T1	-	-	-	-	-	-	-
	T2	67.4 ± 5.6b	73.2 ± 3.7b	70.3 ± 2.1c	70.9 ± 3.7b	68.5 ± 4.4b	11.1 ± 1.4c	9.8 ± 4.2c
	T3	100a	100a	95.7 ± 1.8a	68.5 ± 5.3a	68.3 ± 2.9b	37.5 ± 4.1b	30.8 ± 2.2b
	T4	100a	100a	82.7 ± 2.5b	87.6 ± 6.1a	85.2 ± 7.7a	83.4 ± 5.7a	61.3 ± 7.3a

Values in the same column followed by different letters are significantly different at *P* ≤ 0.05

### Tobacco yield and output value

Due to the outbreak of tobacco bacterial wilt 60 days after transplanting in 2017, the yields of all treatments were zero (Table 2). In 2018, the tobacco yield and the output value of T4 significantly increased compared with each treatment; in T4, yields were 2.71-, 0.61-, and 0.13-fold and output values were 2.44-, 1.62-, and 0.87-fold higher than those of T1, T2, and T3, respectively. In 2019, T4 had the highest tobacco yield and output value, whereas these values were significantly higher in T3 than in T1 and T2, the latter two of which had similar values for these parameters.

**Table 2:** Tobacco yields and output values for the different treatments

Treatment	Tobacco yield (kg hm^−2^)			Tobacco output value (Yuan hm^−2^)		
	2017	2018	2019	2017	2018	2019
T1	0	358.52d	579.14c	0	5905.01d	4808.46c
T2	0	823.27c	618.28c	0	11,015.47c	5227.83c
T3	0	1178.49b	853.76b	0	15,478.76b	11,438.39b
T4	0	1328.81a	1241.95a	0	20,319.54a	19,203.27a

Values in the same column followed by different letters are significantly different at *P* ≤ 0.05

### A dynamic change in pathogen and antagonist counts

**Fig. 1:**
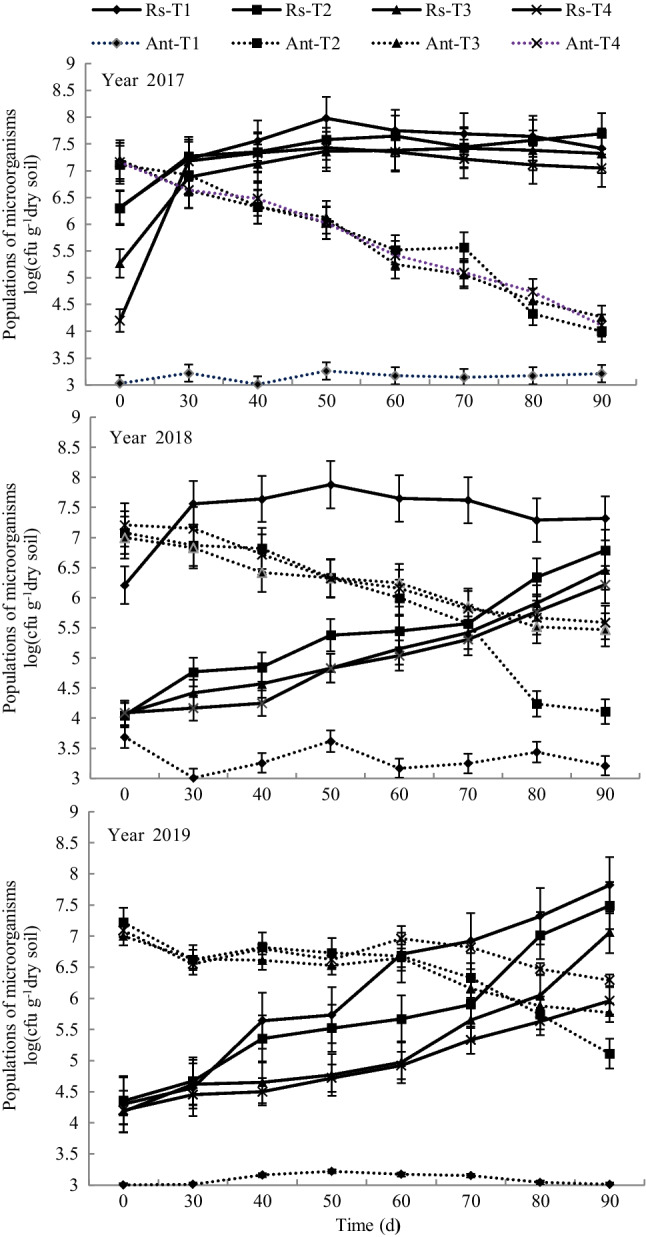
Populations of pathogens and antagonistic bacteria in the soil by year. Note: Rs-T1, tobacco bacterial wilt (*Ralstonia solanacearum* (Rs)) in treatment T1; Rs-T2, Rs in treatment T2; Rs-T3, Rs in treatment T3; Rs-T4, Rs in treatment T4; Ant-T1, antagonistic bacteria in treatment T1; Ant-T2, antagonistic bacteria in treatment T2; Ant-T3, antagonistic bacteria in treatment T3; and Ant-T4, antagonistic bacteria in treatment T4

After 1 year of remediation (2017) and 30 days after transplanting, the soil pathogen population in all treatments remained high at a level above 10^7^ copies/g of soil (Fig. 1), while the counts of antagonistic bacteria in T2, T3, and T4 decreased sharply over the course of the growing period, falling eventually to 10^4^ copies/g soil.

In 2018, after 2 years of remediation, although the populations of soil pathogens in the treatments amended with bioorganic fertilizer had gradually increased, the counts of antagonistic bacteria in the treatments were higher than those of the pathogens during the initial 70 days after transplanting; notably, the pathogen count reached 10^7^ copies/g of soil 30 days after transplanting. Furthermore, 80 days after transplanting, there were no significant differences in the counts of pathogen and antagonistic bacteria between the T3 and the T4 treatments, whereas the population of pathogens was 1.49-fold as high as the population of antagonistic bacteria in T2.

In 2019, all treatments showed a gradual increasing trend in pathogen populations. Fifty days after transplanting, the pathogen population in T1 had sharply increased, while the pathogen population in T2 did not increase until 70 days after transplanting. The population of pathogens in the T4 treatment always remained below 10^6^ copies/g of soil. However, the pathogen counts in the other treatments increased beyond 10^7^ copies/g of soil 90 days after transplanting and afterwards for the duration of the experiment. The antagonistic bacterial counts showed a gradual downward trend during the entire tobacco growing period. Moreover, the pathogen and the antagonistic bacteria counts intersected in T2 at 72 days, while they intersected in T3 at 78 days. However, in T4, 90 days after transplanting, the antagonistic bacteria population was still higher than the pathogen population, reaching 1.23 × 10^6^ copies/g of soil. The antagonistic bacteria count changed from a sharp decrease to a gradual decline over.

### Bacterial community structure in the rhizosphere soil

We compared the α-diversity of microbiota between treatments using the Chao1, Shannon, and Simpson indexes (Table 3). The Chao1 index was highest in T1 treatment, indicating that after soil bioremediation, the total number of microbial species in the soil is relatively reduced. It was found that Shannon and Simpson indexes showed significant difference among treatments. The T4 sample had a significantly (*P* ≤ 0.05) higher α-diversity.

**Table 3:** Alpha diversity index of soil microbiota in different treatments

Treatments	Chao1	Shannon	Simpson
T1	4517.33 ± 70.68a	6.62 ± 0.15c	0.99 ± 0.01b
T2	4375.33 ± 58.32b	6.54 ± 0.10c	0.99 ± 0.00ab
T3	4474.67 ± 76.17ab	6.85 ± 0.07b	0.99 ± 0.00ab
T4	4431.67 ± 19.40ab	7.18 ± 0.02a	1.00 ± 0.00a

Values in the same column followed by different letters are significantly different at *P* ≤ 0.05

**Fig. 2:**
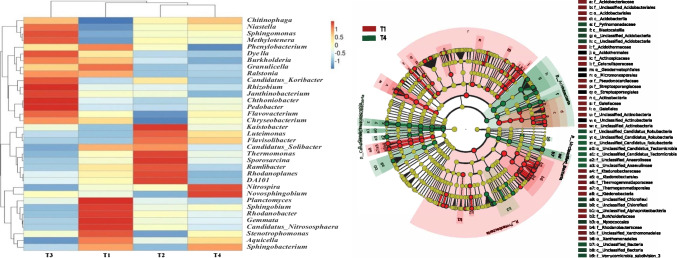
Difference of bacterial community structure composition in rhizosphere soil under different treatments. **a** Species abundance cluster map. Color scale represents the minimum to maximum range of relative abundance of species to be mapped onto the − 1 to 1 input range of the color scale; **b** LEfSe analysis to explore the difference in microbial community structure between treatments. Note: T1, conventional fertilization; T2, conventional fertilization + liming; T3, conventional fertilization + bioorganic fertilizer; T4, conventional fertilization + liming + bioorganic fertilizer

The relative abundance of the top thirty-five soil microorganisms in different treatments was clustered after high-throughput sequencing. As shown in Fig. 2, the dominant taxa significantly differed in the soil community in each treatment. In T1, six taxa—*Planctomyces*, *Sphingobium*, *Rhodanobacter*, *Gemmata*, *Candidatus_Nitrososphaera*, and *Stenotrophomonas*—were relatively abundant and therefore the dominant taxa. The genera *Chitinophaga*, *Niastella*, and *Methylotenera* were notably less abundant in T1 in relation to the other treatments. The taxa *Nitrospira* and *Novosphingobium* were notably more abundant in T4, while the genus *Phenylobacterium* was the least abundant among all treatments. In the T4 treatment, *Nitrospira* and *Novosphingobium* were the most dominant flora, and the dominant genera were significantly higher in the other treatments. The distribution of all the taxa was quite similar, suggesting that the overall bacterial community maintained a consistent balance. Similar patterns of bacterial community structure were observed within the T2 and the T4 treatments. However, no single taxon was dominant across all samples. The genus *Sphingobacterium* showed a similar community structure in the T1 and the T4 treatments, whereas other taxa showed the opposite patterns from these two treatments. Likewise, the taxa in the T2 and the T3 treatments differed from those of T1.

In addition, pathogen of the genus *Ralstonia* was suppressed in T4 and T2 compared with T1. Some of the functional bacteria varied in their distribution among treatments. For example, the genus *Nitrospira*, considered a nitrifier, was much more abundant in T4 than in T1.

LEfSe analysis showed that in phylum, the abundance of the bacterial phylum Proteobacteria was significantly higher in T1 than in T4 treatment, and the abundance of the Xanthomonadales in the bacterial phylum Proteobacteria was extremely high in T1. In bacterial class, the abundance of Actinobacteria and Ktedonobacteria in T1 was also significantly higher than in T4. In contrast, the phylum abundance of Rokubacteria and Tectomicrobia was significantly higher in T4 than in T1, and the class abundance of Chloroflexi in T4 was also significantly higher in T4, compared with T1. It is worth noting that in the bacterial phylum Acidobacteria, the abundance of Acidobacteria and Blastocatellia in T4 was significantly higher than that of T1, while the abundance of bacteria Acidobacteriia in T4 was significantly lower than that of T1.

**Fig. 3:**
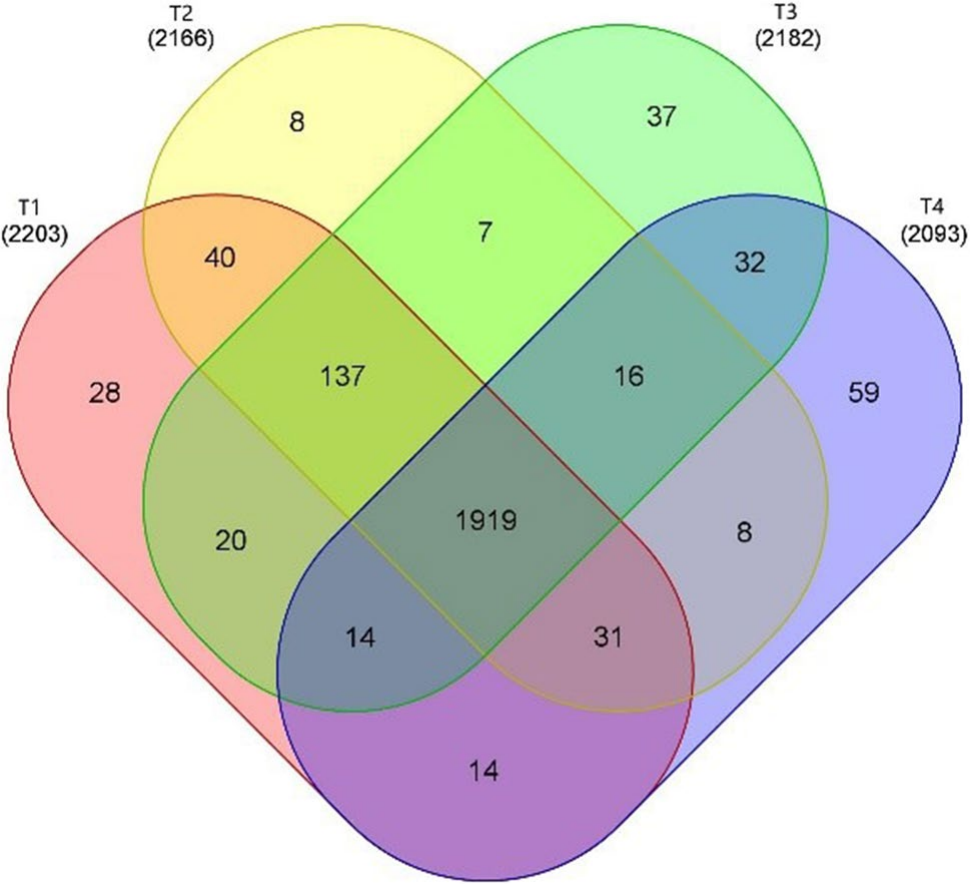
Venn diagram of genus abundance in soil of different field treatments. Note: T1, conventional fertilization; T2, conventional fertilization + liming; T3, conventional fertilization + bioorganic fertilizer; T4, conventional fertilization + liming + bioorganic fertilizer

After the integrated biocontrol of the tobacco bacterial wilt, the number of observed OTUs was lower in T2, T3, and T4 than that in T1 (Fig. 3). The unique OTU number was 2.1 times in T4 of that in T1. The unique OTU number accounted for 1.27%, 0.37%, 1.70%, and 2.82% for T1, T2, T3, and T4 of the total bacterial communities, respectively.

**Fig. 4:**
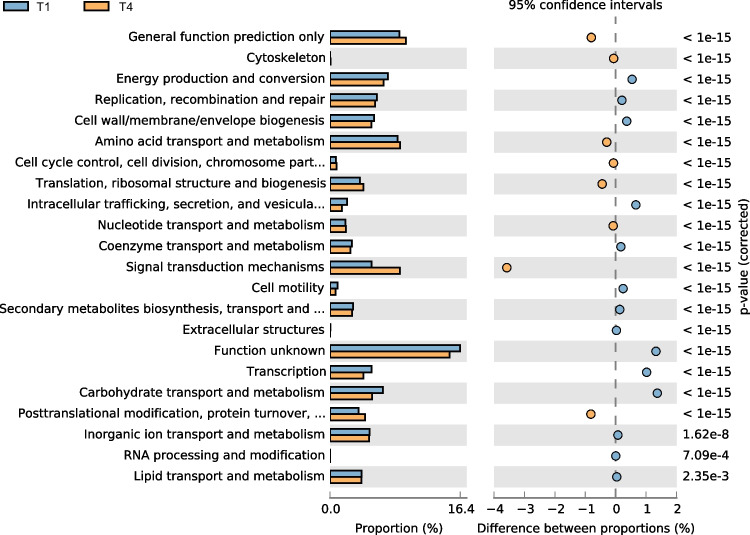
Clusters of orthologous groups of proteins (COG) of soils before and after bioremediation. Note: T1: conventional fertilization; T4: conventional fertilization + liming + bioorganic fertilizer

The cluster of orthologous groups of proteins (COG) was employed to annotate the genomes of soil microorganisms. The T1 treatment and T4 treatment shared some typically bacterial proteins, such as nucleotide transport and metabolism (Fig. 4). However, there were many differences in carbohydrate transport and metabolism, signal transduction mechanisms, and transcription between T1 and T4 treatment. In particular, the abundance of energy production and conversion, replication, recombination and repair, and cell wall/membrane/envelope biogenesis were significantly high in T1 treatment, compared with T4 treatment. Among all these differentially predicted genes, 8 gene abundances of T4 treatment including signal transduction mechanisms were increased while 14 gene abundances were decreased, compared with those of T1 treatment.

**Fig. 5:**
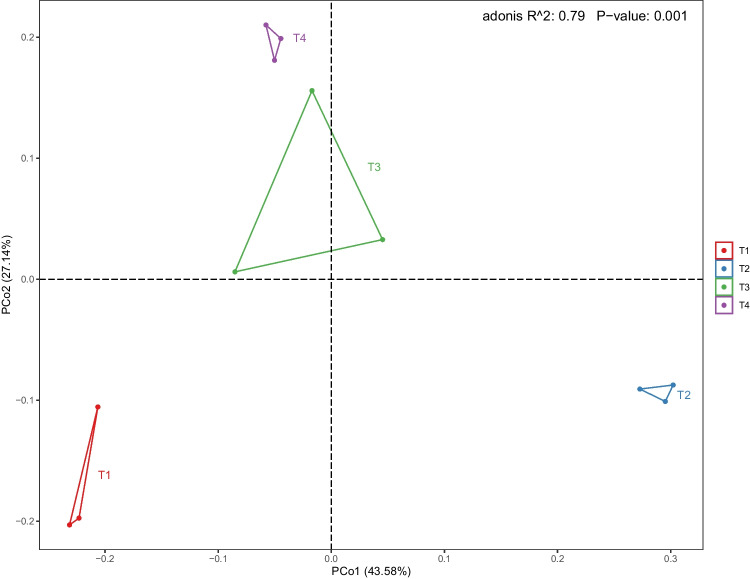
Principal component analysis (PCA) of beta diversity based on classification of the OTUs at a dissimilarity level of 0.03 for individual samples relative to the field treatments. Note: T1, conventional fertilization; T2, conventional fertilization + liming; T3, conventional fertilization + bioorganic fertilizer; T4, conventional fertilization + liming + bioorganic fertilizer

Principal coordinates analysis (PCoA) is a statistical procedure that uses non-binding data dimensionality reduction analysis method, which presents the visual coordinates of the similarity or difference of the study data. PCoA clearly presented the community composition variation among treatments (Fig. 5). The first and second principal components explained 70.72% of the total bacterial community variation among individual samples. Concomitantly, based on the analysis of β-diversity, among the treatments, T3 and T4 shared a similar bacterial assemblage and thus are grouped in the same area. The OTUs shifted greatly between T2, T3, and T4 along the first and second principal component axes. Moreover, T1 was isolated from the T2 treatment along the first principal component axis, while T1 was isolated from T3 and T4 treatments along the second principal component axis, indicating that the bacterial community structure of T1 was significantly different (*P* ≤ 0.01) from those of the other treatments, but the contribution of the two axes is distinct.

**Fig. 6:**
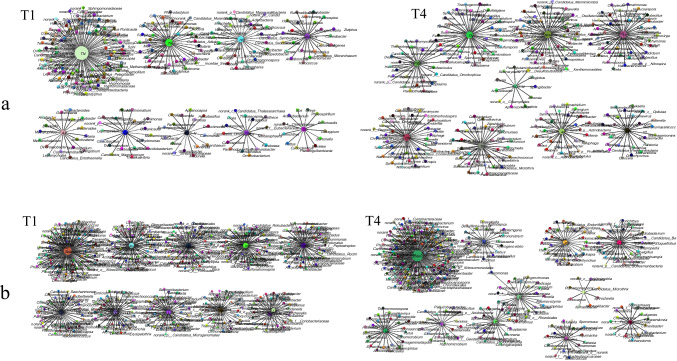
Correlation of soil microflora and character indexes between treatment T1 and T4. Note: **a** Correlation of soil microflora and physicochemical properties. **b** Correlation of soil microflora and tobacco leaf quality indicators. T1, conventional fertilization; T4, conventional fertilization + liming + bioorganic fertilizer

The correlation between microbial genus and soil variables is showed in Fig. 6a. In treatment T4, the size of the constructed genus for total nitrogen (TN) was 39, of which 33 genera positively correlated and 6 genera negatively correlated. However, in T1 treatment, 13 genera positively correlated with TN, while 85 negatively correlated with TN. These data suggested that the node composition of two networks was not similar. The module-trait relationship analysis indicated that the genera in T1 treatment were highly correlated with TN, while the genera in T4 treatment were evenly correlated with soil physicochemical properties. Soil bioremediation enhanced the connection between soil microorganisms and environmental factors, thus forming a more interactive and relevant micro-system. Besides, *R. solanacearum* was positively correlated with total potassium (AK) and NO_3_-N in T1 treatment while *R. solanacearum* was negatively correlated with AK and NO_3_-N in T4 treatment.

The microbial genera related to tobacco leaf quality indicators in the T4 treatment were 1.34% higher than that in the control soil (Fig. 6b). In control treatment, it was a positive correlation between *R. solanacearum* and nicotine, total nitrogen (LTN), solanone, solanedione (Ns), and disease index (DI). The correlation coefficients were 0.7109, 0.4890, 0.9932, 0.7392, and 0.9764, respectively. While *R. solanacearum* and total sugar (TS), reducing sugar (RS), potassium (LK), megastigmatrienone (Meg), tobacco yield (Y), and tobacco output value (V) had a negative correlation. The correlation coefficients were − 0.6390, − 0.5291, − 0.9350, − 0.7641, − 0.9471, and − 0.9342, respectively. In addition, the significant positive correlation (*P* ≤ 0.01) between the microorganisms in the control soil and the tobacco leaf quality factors was greater than the significant negative correlation (*P* ≤ 0.01), while the significant negative correlation between the microorganisms in the treated soil and the tobacco leaf quality factors was much greater than the significant positive correlation, indicating that treatment T4 reduced the effect of soil microorganisms on tobacco leaf quality. Tobacco leaf quality was more likely to remain stable after soil microbial improvement. Compared with the control soil, the significant correlation between soil microorganisms and tobacco solanone in the treated soil was enhanced with 19.3%.

*Alteromonas* had a significant correlation with both nicotine and tobacco output value. Genus such as *Cynara*, *Anditaea*, *Parvimonas*, *Trichosporon*, *Mycosphaerella*, *Acidiphilium*, *Jeotgalibaca*, *Gilvimarinus*, and other microorganisms had significant correlations with leaf yield and total sugar content of tobacco leaves. However, in the control treatment, tobacco leaf quality and soil microorganisms were independent and uncorrelated with each other. As for *Nitrospira*, a microbial genus with high abundance in T4 treatment, it had positively correlation with total soil phosphorus, which was proved to impact the aroma of tobacco leaves. The dominant microorganisms from LEfSe analysis had a significant positive correlation with the quality characteristics of tobacco leaves and physicochemical properties of soil. For instance, *Rokubacteria* had a significantly positive regulation with leaf solanedione and soil NH_4_^+^-N. Likewise, *Acidobacteriales* positively regulated leaf nicotine and potassium content.

Furthermore, the relationship between the microorganisms in the control soil and the tobacco or soil varieties was one-to-one association, while the correlation between the microorganisms in the treated soil and the environmental factors was cross-interacting. The *Symbiobacterium*, Burkholderia, *Chelatococcus*, *Pontibacillus*, and *Caldicellulosirup* were considered as the potential keystone taxa in regulating soil characteristics, since these genera were the shared nodes in the co-occurrence networks. Likewise, the *Alteromonas*, *Anditalea*, *Cynara*, etc. were considered as the potential keystone taxa in regulating leaf quality characteristics. A comprehensive view of the network between soil microbes and physicochemical traits or tobacco leaf quality traits suggested that after soil bioremediation, microorganisms had a strong regulatory effect on the quality of soil and tobacco leaves.

## Discussion

### Suppress the population of pathogen to control tobacco bacterial wilt

The control efficacy in the treatments increased initially and then decreased during the tobacco growing season. Soil bioremediation measures suppressed and delayed the outbreak of the tobacco bacterial wilt in the preliminary growing stage. The onset of tobacco bacterial wilt in this trial was delayed by 30 days (the second year) and 40 days (the third year) with the integrated control measures treatment. In comparison, the control efficacy early in the growing season in T3 and T4 was higher in 2019 than in 2018. Moreover, early in the growing season, the control efficacy in these treatments in 2019 was higher than that in 2018, while the control efficacy in these treatments late in the growing season in 2019 was lower than that in 2018. This difference in 2018 occurred because of high temperature (> 30 °C) and high humidity (> 80%) at 30 days after transplanting, which was the same time the tobacco bacterial wilt occurred. During that time, the pathogen population counts of the T3 and the T4 treatments showed a gradual increasing trend, whereas the counts of antagonistic microorganisms were significantly higher than the pathogen counts 70 days after transplanting; at 90 days after transplanting, the pathogen population failed to reach 10^7^ copies/g of soil (Liu et al. 2014). As a consequence, the control efficacy was greater than 60%. However, there was continuous hot and wet weather 70 days after transplanting in 2019, when the antagonist microorganism counts in T3 and T4 treatments decreased to a level equal to or less than the pathogen counts, which were 10^7^ copies/g of soil. Under these conditions, tobacco bacterial wilt spread in the field. Therefore, in 2019, the control efficacy in T3 and T4 was relatively high early in the growing season and declined late in the growing season. The combination application of bioorganic fertilizer and agronomic measures has a unique advantage that the pathogen population on the root surface and in the rhizosphere was suppressed to less than 10^7^ copies/g, which is the tipping point of pathogens inducing wilt disease. Keeping the pathogen population below the number of critical point is one of the most crucial goals of biocontrol (Zhao et al. 2011).

The soil bioremediation, consisting of liming and bioorganic fertilizer, has effectively controlled various soil-borne diseases (Trillas. et al. 2006). In this trial, compared with the T1 treatment (CK), the control efficacies of the bacterial wilt in the T4 treatment (integrated control measures) reached 75.2% (the second year) and 61.3% (the third year). On one hand, soil pH correction through liming to neutralize the acidity to levels amenable for plant growth is an important agricultural practice for improved productivity. On the other hand, bioorganic fertilizer, the antagonistic bacteria added with a nutrient carrier, consequently prevents *R. solanacearum* from colonizing root niches in the early stage of the tobacco growing season (El-Abyad et al. 1993). However, a limited effect on efficiently controlling tobacco bacterial wilt has been achieved either by single liming or by bioorganic fertilizer application. The integration of liming and bioorganic fertilizer in tobacco production not only improves soil texture, thus facilitating deep rooting and enhancing stress resistance (Jiang et al. 2017), but also decreases the pathogen population (Wang et al. 2013b).

### Improve soil microbial community structure and function to remediate soil

The efficient achievements of disease control mainly depend on the microbial community structure. Different patterns of community structure were observed between control soil and bioremediated soil in our study. In particular, high relative abundances of *Ralstonia*, *Candidatus_Solibactor*, *Stenotrophomonas*, and etc. were observed in control, while *Chitinophaga*, *Nitrospira*, etc. were richer in T4. With a bacterial wilt disease incidence of 100% in the field, the abundance of *Ralstonia* is highest in control among treatments. *Ralstonia* is the pathogen that caused Solanaceae crop’s bacterial wilt. After soil remediation, the improvement of soil microbial community structure, especially the abundance of *Ralstonia*, was significantly reduced, so that the dominant position of pathogenic bacteria was reduced, and the pathogen population was less than the tipping point inducing wilt disease (Li et al. 2017a, b). *Candidatus_Solibactor* belongs to Acidobacteria, which is sensitive to soil pH and was negatively correlated with soil pH (Rousk et al. 2010). Elevated pH is detrimental to the survival of *Ralstonia* in the soil (Gu et al. 2016). Thus, the disease index was significantly reduced. *Chitinophaga* are strongly chitinolytic and can decompose fungal cell walls, thus considered as antagonistic bacteria to fungal pathogens. Besides, *Nitrospira* is a kind of nitrite-oxidizing bacteria, and are members of a distinct phylum, not closely related to other nitrifiers (Frank Maixner et al. 2008). Besides, via the nutrient competition, diverse beneficial communities have significantly stronger ability to resist the invasion of pathogenic bacteria (Liu et al. 2023). Therefore, we hypothesize that various microbial community structures reflect different soil environmental situations. These results were consistent with other studies performed by Brockett et al. (2012), who demonstrated that both structure and enzyme activities of soil microbial communities significantly separated along the regional climate gradient, despite high local variation.

According to the COG results of soil microorganism before and after bioremediation, energy production and conversion, replication, recombination and repair, and cell wall/membrane/envelope biogenesis, most of which belonged to the quorum sensing genes, were all related to plant defense against pathogenic bacteria (Dang et al. 2020). After bioremediation, the soil has reduced energy or function to defend against pathogenic bacteria, indicating that the soil is transitioning from an unhealthy or sub-healthy state to a healthy state.

### Correlation of soil microflora and character indexes

In this study, we demonstrated that the genera were highly correlated with total nitrogen in control, while the genera were evenly correlated with soil physicochemical properties in T4 treatment. If the microorganisms are closely related to a single physicochemical trait, then the micro-ecosystem will be affected by this physicochemical trait and have poor stability (Liu et al. 2021). Otherwise, if the microorganisms are associated with a variety of physical and chemical traits, forming an association network, then the microbial ecosystem will not be fragile and keep stable (Wang et al. 2017). Our results were similar with some reports that showed several soil properties such as soil available P, available K, and TN and the C/N ratio were significantly correlated with abundant phyla (Zhao et al. 2014a). This finding may be a good explanation for soil microbial balance, which was also proved by other research that the application of integrated bioorganic fertilizers effectively improved the soil microbial balance and restored the soil ecosystem, resulting in a complex and healthy soil microbial system (Liu et al. 2013). As such, we hypothesize that soil well-structured bacterial community can improve physical–chemical properties. These findings support the previously tested measures for controlling soil-borne diseases (Zhang et al. 2008).

In our study, we found that the bacterial communities of the T3 and the T4 treatments were clustered together based on the principal component analysis, indicating a similar soil microbial structure and functional diversity. In contrast, the T1 treatment was separated from the T4 treatment in the PCA, indicating distinct bacterial communities. These results were consistent with other studies performed by Zhang et al. (2008) and Lang et al. (2012). Most of the variation that determine the community group can be explained by the interactions among soil properties, such as total nitrogen, total phosphorus, total potassium, and available nitrogen and organic matters. As previously reported, when the soil properties, the microorganism ecology, and the microorganism activity are improved, the soil microbial structure and functional diversity support healthier plants (Zhang et al. 2012; Luo et al. 2015). As for the interactions of species in the co-occurrence networks, we were able to find that the microbiomes in T4 were associated with large number of nodes and edges compared to that in T1 treatment, indicating that the soil was transitioned from diseased soil to healthy soil (Wei et al. 2019). After the soil remediated, the keystone species play different roles compared with the original soil, thus signifying the complexity of multi-species interactions and achieving a closely relevant micro-system, which was ecologically meaningful to the environment (Wang et al. 2017). The linked members in a module were functionally associated taxa that work together to achieve a distinct function or an ecological process. In this research, the integrated control measures, comprising a combination of bioorganic fertilizer application and liming, not only can effectively control tobacco bacterial wilt and significantly improve the flue-cured tobacco yield and output value but can also in general assist in recovering the microbial community, representing a promising application whose utility might be extended to other crops. It is a breakthrough that role-shifts prevailed among the network members. Microbes were unipathically associated with variables in control but multiplex in bioremediated soil. These measures can act to transform a wilt-inducing soil to a healthy and fertile soil, thus efficiently enabling the biocontrol of tobacco bacterial wilt in severely affected fields.

### Supplementary Information

Below is the link to the electronic supplementary material.

Supplementary file1 (PDF 328 KB)

## Data Availability

The data that support the findings of this study are available from the corresponding author upon reasonable request. The sequence of *R. solanacearum* that supports the findings of this study has been deposited in GenBank with the accession code of KC888020; the screened high-efficiency antagonistic bacteria strain LX5 has been deposited in the China General Microbiological Culture Collection Center with the accession code of CGMCC 8263.

## References

[CR1] Abawi GS, Widmer TL (2000). Impact of soil health management practices on soilborne pathogens, nematodes and root diseases of vegetable crops. Appl Soil Ecol.

[CR2] Almeida E, Serra CR, Albuquerque P, Guerreiro I, Teles AO, Enes P, Tavares F (2018). Multiplex PCR identification and culture-independent quantification of *Bacillus licheniformis* by qPCR using specific DNA markers. Food Microbiol.

[CR3] Bao SD (2000). Soil agrochemical analysis.

[CR4] Brockett B, Prescott C, Grayston S (2012). Soil moisture is the major factor influencing microbial community structure and enzyme activities across seven biogeoclimatic zones in western Canada. Soil Bio Biochem.

[CR5] Carlsen SCK, Pedersen HA, Spliid NH, Fomsgaard IS (2012) Fate in soil of flavonoids released from white clover (*Trifolium repens* L.). Appl Environ Soil Sci 1–10. 10.1155/2012/743413

[CR6] Chen WT (2014) Study and integration on synthetically prevention technology of *Rhizoctonia Solani* from potato. Dissertation, Neimenggu Argicultual University

[CR7] Dang X, Zhang L, Wang W, Wang G, Liu R, Xie Z (2020). Soil microbial community structure along the ecological succession in Yellow River Delta. Acta Microbiol Sin.

[CR8] Deng Y, Jiang YH, Yang Y, He Z, Luo F, Zhou J (2012). Molecular ecological network analyses. BMC Bioinformatics.

[CR9] El-Abyad MS, El-Sayed MA, El-Shanshoury AR, El-Sabbagh SM (1993). Towards the biological control of fungal and bacterial diseases of tomato using antagonistic *Streptomyces* spp. Plant Soil.

[CR10] Gu Y, Hou Y, Huang D, Hao Z, Wang X, Wei Z, Jousset A, Tan S, Xu D, Shen Q, Xu Y, Friman V-P (2016). Application of biochar reduces *Ralstonia solanacearum* infection via effects on pathogen chemotaxis, swarming motility, and root exudate adsorption. Plant Soil.

[CR11] Jiang G, Wei Z, Xu J, Chen H, Zhang Y, She X, Macho AP, Ding W, Liao B (2017). Bacterial wilt in China: history, current status, and future perspectives. Front Plant Sci.

[CR12] King SR, Davis AR, Liu W, Levi A (2008). Grafting for disease resistance. HortScience.

[CR13] Lang J, Hu J, Ran W, Xu Y, Shen Q (2012). Control of cotton *Verticillium* wilt and fungal diversity of rhizosphere soils by bio-organic fertilizer. Biol Fertil Soils.

[CR14] Li S, Gu L, Liu K, Liao Z (2009). Effects of combined application of organic fertilizers on the control of soilborne diseases and the regulation of soil microbial diversity. Plant Nutr Fertil Sci.

[CR15] Li H, Wu HW, Meng FT, Sun LD, Xie ZY, Li LY (2013). Development of a PCR method for rapid detection of *Bacillus licheniformis* based on gyrB gene. Nat Sci J Hainan Univ.

[CR16] Li LY, Xu YM, Wang CD, Chen AG, Tao J, Liu BZ, Zhao P, Wang SS (2016). Analysis of aroma components in flue-cured tobacco leaves in Guizhou tobacco-growing areas. Chinese Tobacco Sci.

[CR17] Li X, Liu XY, Xia FJ, Cai LT, Zhang H, Shi JX (2017). Screening, identification and plant growth-promotion mechanism of tobacco plants rhizobacteria. Acta Tabacaria Sinica.

[CR18] Li X, Liu Y, Cai L, Zhang H, Shi J, Yuan S (2017). Factors affecting the virulence of *Ralstonia solanacearum* and its colonization on tobacco roots. Plant Pathol.

[CR19] Liu D (2012) Study on the production of cullulases and comparative secretome analysis in *Aspergillus fumigatus* Z5 isolated from composting. Dissertation, Nanjing Agricultural University

[CR20] Liu XC (2014) A study on effects of changes and control of temperature and humidity on the occurring of tobacco bacterial wilt and the control techniques. Dissertation, Southwest University

[CR21] Liu Y, Shi J, Feng Y, Yang X, Li X, Shen Q (2013). Tobacco bacterial wilt can be biologically controlled by the application of antagonistic strains in combination with organic fertilizer. Biol Fertil Soils.

[CR22] Liu Y, Li X, Cao Y, Lu N, Shi J (2014). Field control efficiency of tobacco specific bio-organic fertilizer on tobacco bacterial wilt. J Plant Nutr Fertil.

[CR23] Liu Y, Li X, Cai K, Cai L, Lu N, Shi J (2015). Identification of benzoic acid and 3-phenylpropanoic acid in tobacco root exudates and their role in the growth of rhizosphere microorganisms. Appl Soil Ecol.

[CR24] Liu S, Wang Z, Niu J, Dang K, Zhang S, Wang S, Wang Z (2021). Changes in physicochemical properties, enzymatic activities, and the microbial community of soil significantly influence the continuous cropping of Panax quinquefolius L (American ginseng). Plant Soil.

[CR25] Liu Y, Tao Z, Li X, Zhang H, Zhu J, Wang F, Jiao J, Wang K, Xu J, Wang W, Li H (2023). Construction of bacterial wilt-resistant and plant growth-promoting rhizobacteria (PGPR) and the mechanism of biocontrol. Acta Microbiol Sin.

[CR26] Luo J, Liu L, Wang T, Liu H, Yan S, Lu X, Fan R, Zhang Z (2015). Effect of organic fertilizer from deep-litter pig rearing on pepper yield and soil microbial diversity. Soils.

[CR27] Maixner F, Wagner M, Lücker S, Pelletier E, Schmitz-Esser S, Hace K, Spieck E, Konrat R, Le Paslier D, Daims H (2008). Environmental genomics reveals a functional chlorite dismutase in the nitrite-oxidizing bacterium “Candidatus *Nitrospira defluvii*”. Environ Microbiol.

[CR28] Rousk J, Baath E, Brookes P, Lauber C, Lozupone C, Caporaso J, Knight R, Fierer N (2010). Soil bacterial and fungal communities across a pH gradient in an arable soil. ISME J.

[CR29] Trillas MI, Casanova E, Cotxarrera L, Ordovas J, Borrero C, Aviles M (2006). Composts from agricultural waste and the *Trichoderma asperellum* strain T-34 suppress *Rhizoctonia solani* in cucumber seedlings. Biol Control.

[CR30] Wang BB, Yuan J, Zhang J (2013). Effects of novel bioorganic fertilizer produced by *Bacillus amyloliquefaciens* W19 on antagonism of *Fusarium* wilt of banana. Biol Fert of Soils.

[CR31] Wang L, Shi J, Yuan S, Wu K, Cai L, Liu Y, Yang X, Feng Y, Shen B, Shen Q (2013). Control of tobacco bacterial wilt with biomanure plus soil amendments. Acta Pedol Sin.

[CR32] Wang H, Wei Z, Mei L, Gu J, Yin S, Faust K, Raes J, Deng Y, Wang Y, Shen Q, Yin S (2017). Combined use of network inference tools identifies ecologically meaningful bacterial associations in a paddy soil. Soil Biol Biochem.

[CR33] Wei Z, Huang J, Yang T, Jousset A, Xu Y, Shen Q (2017). Seasonal variation in the biocontrol efficiency of bacterial wilt is driven by temperature-mediated changes in bacterial competitive interactions. J Appl Ecol.

[CR34] Wei Z, Gu Y, Friman V, Kowalchunk G, Xu Y, Shen Q, Jousset A (2019). Initial soil microbiome composition and functioning predetermine future plant health. Sci Adv.

[CR35] Wu K, Yuan S, Wang L, Shi J, Zhao J, Shen B, Shen Q (2014). Effects of bio-organic fertilizer plus soil amendment on the control of tobacco bacterial wilt and composition of soil bacterial communities. Biol Fertil Soils.

[CR36] Zhang Z, Li H, Wei X, Liu X, Peng G (2008). Influence of biological fertilizers on banana wilt disease and microorganisms in soil. Ecol Environ.

[CR37] Zhang N, Wu K, He X, Li S, Zhang Z, Shen B, Yang X (2011). A new bioorganic fertilizer can effectively control banana wilt by strong colonization with *Bacillus subtilis* N11. Plant Soil.

[CR38] Zhang X, Mao J, Li Z, Li G, Wang M, Jiang K, Wang H, Duan H, Li Y (2012). Effect of nitrogen fertilizer rates and ratios of base and topdressing fertilizer on yield, quality of tobacco leaves and N-use efficiency. Plant Nutr Fertil Sci.

[CR39] Zhao Q, Dong C, Yang X, Mei X, Ran W, Shen Q, Xu Y (2011). Biocontrol of *Fusarium* wilt disease for *Cucumis melo* melon using bio-organic fertilized. Appl Soil Ecol.

[CR40] Zhao J, Zhang R, Xue C, Xun W, Sun L, Xu Y, Shen Q (2014). Pyrosequencing reveals contrasting soil bacterial diversity and community structure of two main winter wheat cropping systems in China. Soil Microbiol.

[CR41] Zhao S, Liu D, Ling N, Chen F, Fang W, Shen Q (2014). Bio-organic fertilizer application significantly reduces the *Fusarium oxysporum* population and alters the composition of fungi communities of watermelon *Fusarium* wilt rhizosphere soil. Biol Fertil Soils.

[CR42] Zhou J, Deng Y, Luo F, He Z, Yang Y (2011) Phylogenetic molecular ecological network of soil microbial communities in response to elevated CO_2_. mBio 2:e00122–00111 10.1128/mBio.00122-11 PMC3143843 21791581

