# Supplementary material for: Effect of soil bioremediation on soil microbial community structure aimed at controlling tobacco bacterial wilt
Source: Appl Microbiol Biotechnol. 2023 Sep 27;107(24):7543–55. doi: 10.1007/s00253-023-12753-4 (PMC10656326; doi:10.1007/s00253-023-12753-4)
Supplement: Supplementary file 1 — Supplementary file1 (PDF 328 KB) [file 253_2023_12753_MOESM1_ESM.pdf]

Journal Name: Applied Microbiology and Biotechnology

Manuscript Title: Effect of Soil Bioremediation on Soil Microbial Community Structure Aimed at Controlling Tobacco Bacterial Wilt

The names of the authors: Yanxia LIU[1&], Han LI[1&], Xiang LI [2*], Heng ZHANG [1*], Jingwei ZHU[1], Yu PENG[2], Guangjun, SUN[2], Jian XU[2]

[&]These authors contributed to the work equally and should be regarded as co-first authors.

Corresponding author: LI Xiang*, E-mail: newcool1361214@163.com, Tel: +86-18685188016

The affiliations and addresses of the authors: 1. Guizhou Academy of Tobacco Science (Guizhou Provincial Academician Workstation of Microbiology and Health), No. 29, Longtanba Road, Guanshanhu District, Guiyang, Guizhou Province, China, 550000

2. Guizhou Tobacco Corporation of CNTC, No. 146, North Ruijin Road, Guiyang, Guizhou Province, China, 550000

The e-mail address, telephone and fax numbers of the corresponding author: E-mail: newcool1361214@163.com, Tel: +86-18685188016, fax number: 0851-84116909

Table and figure captions

Table S1 Microbial counts before and after secondary fermentation of antagonistic bacteria (cfu/g of fertilizer)

Table S2 Soil physi-chemical properties of different treatments

Table S3 Tobacco quality index of different treatments

Fig. S1 The effect of integrated control measures on tobacco bacterial wilt

Note: T1: conventional fertilization; T4: conventional fertilization+liming+bioorganic fertilizer

The supplementary tables

Table S1 Microbial counts before and after secondary fermentation of antagonistic bacteria (cfu/g of fertilizer)

Fertilizer Treatments	Bacteria	Actinomycetes	Fungi	Antagonists
Organic fertilizer (OF, before secondary solid fermentation)	1.31±0.21× 10^8b	1.27±0.35× 10^5b	1.94±0.08× 10^5a	4.12±0.21× 10^3b
Strain LX5 bioorganic fertilizer (BOF_5, after secondary solid fermentation)	1.91±0.48× 10^9a	2.12±0.11× 10^8a	2.07±0.74× 10^5a	1.91±0.55× 10^9a

Note: Values in the same column followed by different letters are significantly different at *P≤0.05*.

Table S2 Soil physi-chemical properties of different treatments

Treatment	Total Nitrogen TN(%)	Alkeline-N HN(mg/kg)	Total Phosphorus TP(g/kg)	Available Phosphorus AP(mg/kg)	Total Potassium (%)	Available Potassium AK(mg/kg)	Organic Matter OM(g/kg)	NH^{4+}-N (mg/kg)	NO_3^--N (mg/kg)
T1	0.19a	145.94d	0.98b	38.10b	1.74b	234.75b	33.51c	22.09c	27.30c
T2	0.18b	175.43c	0.84c	29.77c	1.73b	156.67c	34.02c	18.20d	28.33a
T3	0.20a	196.60b	0.92b	36.51b	1.78a	225.82b	37.19b	25.77b	28.02b
T4	0.20a	285.93a	1.11a	53.36a	1.78a	260.81a	39.86a	26.45a	28.36a

Note: Values in the same column followed by different letters are significantly different at *P≤0.05*.

Table S3 Tobacco quality index of different treatments

Treatment	Nicotine (%)	Total Sugar (%)	Reducing Sugar (RS)%	Total Nitrogen (TN) %	Potassium (LK)%	Megastigmatrienone（Meg）μg/g	Solanone μg/g	Norsolandione (Ns) μg/g
T1	3.52a	19.52c	16.85c	2.85a	2.20b	8.26c	4.36c	3.30c
T2	3.50a	19.33d	16.49d	2.89a	2.15c	8.27c	4.39c	3.32c
T3	3.43b	20.16b	17.06b	2.89a	2.22b	8.46b	4.61b	3.49b
T4	3.33c	20.89a	17.67a	2.63b	2.53a	8.58a	4.86a	3.58a

Note: Values in the same column followed by different letters are significantly different at *P≤0.05*.

The supplementary Figures

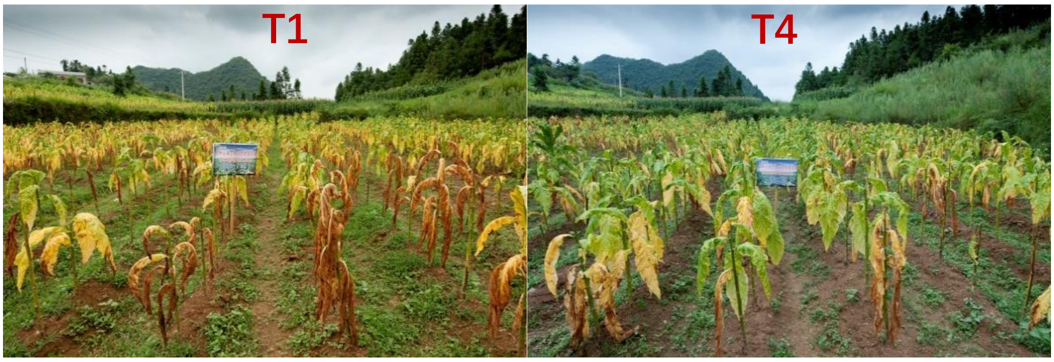

Fig. S1
